# Chromosomal Instability in Genome Evolution: From Cancer to Macroevolution

**DOI:** 10.3390/biology12050671

**Published:** 2023-04-28

**Authors:** Valentine Comaills, Maikel Castellano-Pozo

**Affiliations:** 1 Andalusian Center for Molecular Biology and Regenerative Medicine—CABIMER, University of Pablo de Olavide—University of Seville—CSIC, Junta de Andalucía, 41092 Seville, Spain; 2 Genetic Department, Faculty of Biology, University of Seville, 41080 Seville, Spain

**Keywords:** chromosomal instability, cancer, genome evolution, speciation, structural variant, meiosis, micronuclei

## Abstract

**Simple Summary:**

The experimental/pathological observation of genome chaos (including massive and large-scale translocations, chromothripsis, and polyploidy cancer cells) highlighted the importance of genome reorganization in evolution. Recent advances in sequencing and bioinformatic analysis have highlighted such chromosomal diversity. The evolution of the genome has been studied in the field of macroevolution and speciation as well as in the context of cancer and tumor progression. Evolution is inherent to adaptation to the environment and preparing for future pressure to initiate survival. Human cells are plastic, and several mechanisms are involved in sporadic timing in view of generating genomic diversity under normal conditions, such as during gametogenesis or during pathology like cancer. Interestingly, patterns of chromosomal instability are strikingly similar in evolution and in cancer. Here we will discuss some events leading to several varieties of chromosomal patterns, from cancer to speciation, and discuss the disease associated with chromosomal instability.

**Abstract:**

The integrity of the genome is crucial for the survival of all living organisms. However, genomes need to adapt to survive certain pressures, and for this purpose use several mechanisms to diversify. Chromosomal instability (CIN) is one of the main mechanisms leading to the creation of genomic heterogeneity by altering the number of chromosomes and changing their structures. In this review, we will discuss the different chromosomal patterns and changes observed in speciation, in evolutional biology as well as during tumor progression. By nature, the human genome shows an induction of diversity during gametogenesis but as well during tumorigenesis that can conclude in drastic changes such as the whole genome doubling to more discrete changes as the complex chromosomal rearrangement chromothripsis. More importantly, changes observed during speciation are strikingly similar to the genomic evolution observed during tumor progression and resistance to therapy. The different origins of CIN will be treated as the importance of double-strand breaks (DSBs) or the consequences of micronuclei. We will also explain the mechanisms behind the controlled DSBs, and recombination of homologous chromosomes observed during meiosis, to explain how errors lead to similar patterns observed during tumorigenesis. Then, we will also list several diseases associated with CIN, resulting in fertility issues, miscarriage, rare genetic diseases, and cancer. Understanding better chromosomal instability as a whole is primordial for the understanding of mechanisms leading to tumor progression.

## 1. Introduction

The genome is organized into chromosomes that provide the structure for the faithful transmission of hereditary information. Maintenance of genome integrity is essential for the survival of all living organisms. To accomplish this, cells have developed intricate DNA repair mechanisms to maintain genome integrity; however, aberrant dynamics of the genome or DNA-damage repair, such as double-strand breaks (DSBs), can lead to genome instability, including chromosomal anomalies and mutations, which can promote tumorigenesis over an individual’s lifetime [1]. Tumorigenesis is the complex evolutionary process of the loss of cell identity and gain of characteristics, with different stages like metastasis and with a base of accumulation of different forms of genome instability. The development of deep sequencing technologies has uncovered new chromosomal abnormalities linked with cancer progression [2], providing new insight into the plasticity and the instability of the genome as well as into the mechanisms underlying the maintenance for modifications of chromosome structure, which provides us a new perspective to understand the instability behind of tumorigenesis. 

Germ cells reflect the evolutionary history and future potential of a species as they give rise to the formation of gametes. Genomic instability in germ cells can lead to chromosomal anomalies, resulting in sterility or genetic diseases in the offspring [3,4,5], but it can also result in stable and heritable rearranged genomic constitutions such as mutations or chromosomal changes [6,7]. Thus, chromosomal instability plays a crucial role in evolution, providing a new advantage for organisms adapting to changing environments. These changes can include changes in chromatin conformation and reorganization to a wide range of factors, single nucleotide polymorphisms, or large-scale structural variants, such as inversions, translocations, fusions, and fissions, which can result in variations in the number and morphology of chromosomes [5,6,8,9]. Over millions of years, the accumulation of these changes can lead to speciation.

Evolution can be divided into two hierarchical processes: microevolution and macroevolution [10]. Microevolution deals with mechanisms that modify allele frequencies in gene pools within a species, such as mutation, migration, genetic drift, and natural selection. These processes accumulate over time and can lead to the emergence of new species through the divergence of populations. In contrast, macroevolution involves evolutionary changes above the species level at a grand scale over long periods, including gradual or rapid changes, adaptive radiations, and extinctions, among others. Macroevolution typically results in the evolution of large-scale structures such as chromosomal rearrangements and can dramatically change the structure and functions of proteins, leading to the evolution of entirely new structures, adaptations, or increased fitness.

This review summarizes the relationship between different sources of genome and chromosome instability associated with cancer, infertility, and evolution, highlighting similar patterns between them, but with a main difference, the scale of time. A proper understanding of the underlying biology will lead to more effective cancer treatment and improved understanding of evolution.

## 2. Different Patterns of Structure Variants and Chromosomal Instability

Chromosomal instability (CIN) is a type of genomic instability in which chromosomes are unstable and is a process resulting in the accumulation of large-scale losses, gains, and rearrangements of DNA. CIN can lead to structural chromosome changes or numerical changes in chromosome numbers.

When CIN occurs in germline cells or during gametogenesis, all the individuals will present a chromosomal variability in their cells that can be stable. In some rare cases, CIN can happen during one of the first mitotic division of early embryologic development and can produce mosaicism. In this case, the individual owns two or more genetically different sets of cells in their body.

The causes of CIN are diverse and include meiosis or mitotic errors, replication stress, homologous recombination deficiency (HRD), telomere crisis, nuclear envelope rupture, breakage-fusion-bridge cycles, and others. When CIN happens in somatic cells, chromosomal instability has complex consequences, including loss or amplification of driver genes, focal rearrangements, extrachromosomal DNA and amplification of oncogenes, micronuclei formation, changes in chromosomal territory, and activation of innate immune signaling [11,12]. Because of the diversity of these causes and consequences, CIN is generally used as an umbrella term [12].

**Figure 1:**
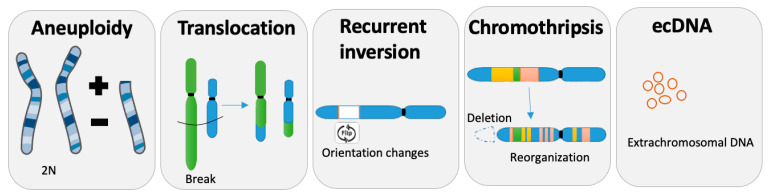
Examples of some structural variants and chromosomal anomalies.

CIN is seen in different forms, more or less complex, and results in structural and numerical chromosomal abnormalities. Some of them are compensated and do not lead to major changes in global expression but can affect some small part of the genome and disrupt some important genes with massive consequences, as in the predisposition to produce cancer during certain translocations or during complex chromosomal rearrangement (Figure 1).

Other terms associated with CIN that need to be defined are CNV, CNA and LOH. CIN can lead to copy number variation (CNV) and copy number alteration (CNA). In general, CNV is a term used in germline cells and CNA in somatic cells.

CNV is a phenomenon in which sections of the genome are repeated, and the number of repeats in the genome varies between individuals. CNV in germline cells is usually non-pathological and represents a variability within a population, and is a type of structural variation. 

In somatic cells and in the case of cancer, CNV has a broader definition as it includes focal changes in localized genomic regions as well as large alterations such as the gain or loss of an entire chromosomal arm and having an abnormal number of chromosomes in a cell. In this case, it is often referred to as CNA. Another term is loss of heterozygosity (LOH), defined as an allelic imbalance because one of the two parental alleles gets lost.

### 2.1. Aneuploidy

The simplest consequence of CIN is the gain or loss of chromosome/s or chromosome arms, named aneuploidy, leading to an unbalanced number of chromosomes (Figure 1). The most common human chromosomal disorder—and the first discovered [13]—is Down syndrome, also referred to as Trisomy 21, where all cells from the body have an extra full or partial [14] chromosome 21. Importantly, aneuploid cells, such as in the case of Down syndrome, can be chromosomally stable and do not result in CIN and a cascade of chromosomal changes. Another extreme case of aneuploidy is the gain in ploidy, where cells can duplicate the entire genome and conclude in a whole genome doubling (WGD). It has been reported that cancer cells can proceed to several rounds of WGD during their progression [15].

### 2.2. Common Chromosomal Changes

The four main types of structural chromosomal aberrations are deletion, duplication, inversion, and translocation (Figure 1). Deletion is the loss of the chromosome in whole or in part. Translocation is when a piece of one chromosome breaks off and attaches to another chromosome. Duplication happens when a part of the chromosome doubles and results in extra genetic material. An inversion in a chromosome occurs when a segment breaks off, turns 180 degrees, and reattaches within the same chromosome. 

### 2.3. Complex Genomic Rearrangements

Structural chromosome abnormalities can also be observed as a complex chromosomal rearrangement (CCR) that involves more than two breakpoints and includes one or more chromosomes. CCRs are not only important causes of fertility issues but are also largely involved in cancer. Chromothripsis, a CCR example, comprises hundreds of chromosomal rearrangements in a localized region of one or few chromosomes, leading to a new configuration of this one [16,17] (Figure 1). Chromoplexy, on the other hand, tends to involve fewer pieces of chromosome but implicates more chromosomes [18]. Other forms of complex genomic rearrangements include breakage-fusion-bridge cycles, which lead to complex duplications and inversions of genomic regions [16]. 

### 2.4. ecDNA 

Extrachromosomal DNA (Figure 1) refers to DNA that is not located on a chromosome but instead exists as a separate, circular entity and invisible using light microscopy but is detectable with biophysical methods or sequencing [19]. ecDNA includes relatively large particles, generally ranging from 1 to 3 Mb, which contain one or multiple full genes and regulatory regions, and they lack a centromere or a telomere [19]. ecDNA is frequently observed across many cancer types and often in high copy numbers and can be associated with the amplifications of several oncogenes, such as *EGFRvIII* [20] or *MYC* [21].

## 3. CIN, Evolution, and Fitness

The creation of genomic diversity is a strategy for preparing for and adapting to new challenges. Macroevolution and speciation have shown the benefits of such events at a large scale. However, CIN’s advantages are not straightforward as it lacks efficiency in offering fitness gain. During the process of generating new offspring in humans, CIN is responsible for high levels of miscarriages since the majority of aneuploid embryos do not develop to term [22]. 

In cancer, such inefficiency is also demonstrated. Aneuploidy also causes general stress in cells due to an imbalanced genome and is often associated with decreased proliferation [23] and imbalanced transcriptome and proteome [24,25]. As such, aneuploidy and chromosomally unstable cells do not offer a direct gain of fitness or advantages to clonal competitions. However, CIN drives tumor progression and is associated with metastasis, drug resistance, and poor prognosis. CIN promotes clonal diversification and intratumoral heterogeneity, giving rise to the genetic diversity that assists tumor progression and aggressiveness [26]. Acquisition of chromosome instability is a mechanism to evade oncogene addiction and offers new options for resisting treatment [27].

Therefore, the more diverse and heterogeneous the population, the greater the odds of adapting, surviving, and resisting. In this sense, it has been proven that the tumor microenvironment plays a crucial role in determining the heterogeneity of tumors [28,29]. This is because of the dynamic and reciprocal interaction between cancer cells and their surroundings. Essentially, a tumor can be viewed as an integrated ecosystem where neoplastic cells co-evolve with various components of the tumor microenvironment, such as the extracellular matrix, immune cells, and tumor vasculature. This results in a wide range of cancer cell phenotype with varying levels of fitness [30]. The “hallmarks of cancer” are different fitness phenotypes or ecological factors that provide selective advantages to cancer cells [28]. These include the ability to sustain proliferative signaling, resist cell death, evading growth suppressors, access vasculature, induce invasion and metastasis, enable replicative immortality, reprogram cellular metabolism, avoid immune destruction, unlock phenotypic plasticity, undergo non-mutational epigenetic reprogramming, harbor polymorphic microbiomes, and house senescent cells [31]. Ultimately, the majority of these hallmarks affect cancer cell fitness by promoting survival and/or reproduction.

## 4. Chromosomal Instability during Evolution

**Figure 2:**
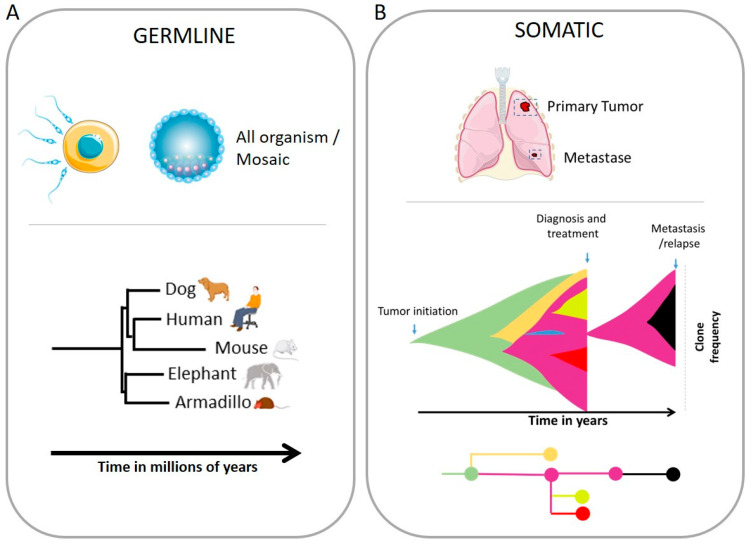
Phylogenetic trees from evolution species (**A**) and from tumors (**B**). (**A**) During evolution, changes in the genome need to be germline, meaning that all cells have the same genetic background, and this genetic is hereditary. For this purpose, those changes need to appear during fecundation or during gametogenesis. All organisms on earth share a common ancestor. Over time, the accumulation of genomic changes led to diversification in specific species. (**B**) Tumor initiation involves changes in the genomes. One cell gains genomic insults, resulting in the formation of tumors. Because of genomic instability, tumor cells derive in diverse clones with their own genetic changes. During tumor progression and metastasis, specific clones will survive in the new environment and under therapeutic pressure.

Adaptive evolution is the ability of genetic changes to adapt to relative fitness through selection. Species modify their genome to survive and diverge into new lineages (Figure 2). To be heritable and then transmitted to the next generation, such changes need to be done through fecundation and the genetic diversification of the gametes. Genome modification can be accomplished through mutations by increasing the genetic diversity or can be more drastic, as observed with changes in structural variants (SV) and the number of chromosomes.

### 4.1. SNPs

The human genome contains 4 to 5 million single nucleotide polymorphisms (SNPs), making the diversity of our species. This genetic variation has specific distribution across the globe [32]. SNPs are present in all species [33].

Because of genomic diversity, species can have different abilities to survive disasters. Point mutations play a major role in creating genomic diversity. Specific genetic variants such as single nucleotide polymorphisms (SNPs) can be crucial in short-term resistance to specific pressures. For example, in the case of the Chornobyl accident and the major release of radioactive material into the environment, it only took 30 years for the frog population to select and shift to a dark-skin coloration population to adapt to the local radiation [34]. Another extreme case is the human response to the Black Death, a pandemic caused by the Y. Pestis infection that devastated Afro-Eurasia, killing up to 30–50% of the human population [35]. Ancient DNA reveals rapid natural selection during this pandemic [35,36]. This study [35] shows that individuals carrying specific genetic immune defense variants had much better odds of surviving infection. In just a few generations, the survivors selected specific variants that responded efficiently to Y. pestis at a speed and an intensity never observed before in human genomes [35,36]. However, the protection afforded by those variants could have come at a cost, as some of those variants have deleterious effects in modern times, as they are associated with auto-immune diseases such as rheumatoid arthritis or Crohn’s disease [35,37]. 

### 4.2. Genome Size Variability and Chromosomal Instability

**Figure 3:**
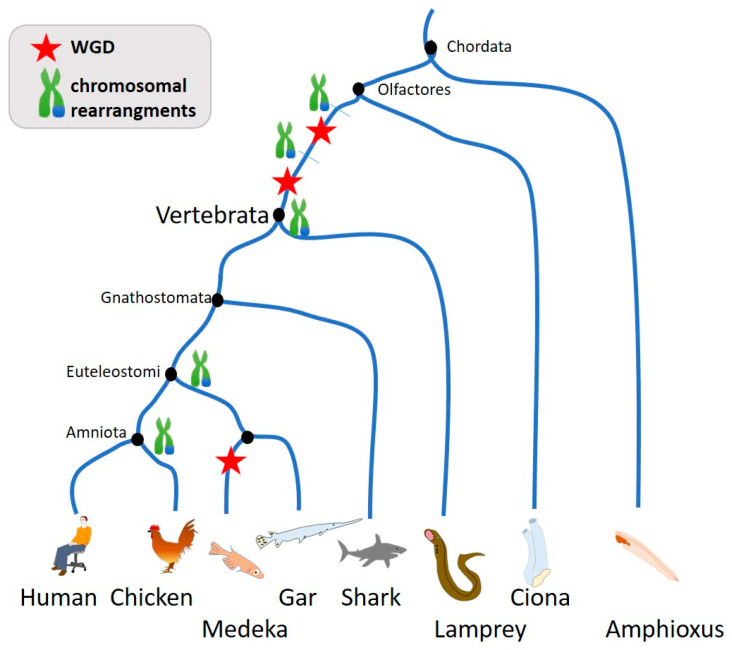
Reconstructed evolutionary history of karyotypes from Chordata to Amniota. A simplified species tree of the Chordata is shown, with WGD events depicted by red stars. The eight lineages represented from left to right are mammals, birds, teleost fish, holostocean fish (gar), cartilaginous fish, cyclostomes (lamprey, hagfish), tunicates (ciona), and cephalochordates (amphioxus). Important events of chromosomal rearrangement such as translocation and fusion are shown (green chromosome with blue arm translocation). Adapted from [39].

Genome size varies greatly over time and supports the diversification and adaptation of the species. In the early time of evolution and diversification, translocation events and primordial whole-genome doubling created such a diversity of life. Recent studies demonstrated the occurrence of two whole-genome duplications in the lineage that preceded the ancestor of vertebrates [38,39] (Figure 3). Some fishes duplicated their genome a third time in view of increasing the genome size [39,40,41,42,43], playing an important evolutionary role in terms of genomic variability and plasticity. However, changes in ploidy do not appear to represent a major source of genome size variation in birds or mammals [40]. Plant kingdoms also present great variability in genome size and WGD events [44].

The gain and loss of some chromosomes are also indispensable during evolution. For example, in the case of the well-studied *Equus* phylogeny over the last 4.5 million years, diversification of at least several dozen recognized genera was observed. Equine phylogeny has shown important SNP but also demonstrates extremely divergent chromosomal structure, both in number (gain and loss) or in the chromosomal rearrangement. As such numbers of chromosomes are really diverse (Horses (*E. caballus*) *n* = 32, Zebras *n* = 16–22, African Ass *n* = 31, and Asian Ass *n* = 26) [45].

Genomic diversity is not only observed as a change in the number of full chromosomes but also involves modification in their structures. Chromosomal structure variants as events of translocation, deletion, duplication, inversion, and complex chromosomal rearrangements, such as chromothripsis (see below), have shaped the history of many evolutionary lineages [39,42,45,46,47].

### 4.3. Chromosomal Variance in Human

Single nucleotide polymorphisms are common in humans and explain the diversity of phenotypes in our species. However, structural variants (SV) also exist in humans and represent 0.1% of variants [48]. In fact, SVs are common in healthy individuals. A study [48] mapping and characterizing the structural variation in 17,795 human genomes has shown that 2% of individuals carry ultra-rare megabase-scale structural variants, nearly half of which are balanced (translocations and inversions) or complex rearrangements. On average, individuals carry 2.9 rare structural variants that alter coding regions and 19.1 rare non-coding deletions; these variants affect the dosage or structure of 4.2 genes [48]. Structural variants in normal individuals are predominantly deletion, mobile-element insertion, and duplication but also present complex rearrangements.

Another study analyzing the complex landscape of 433,371 SVs has shown that SVs are responsible for 25–29% of all rare protein-truncating events per genome. In their study, the authors found 3.9% of individuals carried very large (over one megabase) rare SVs [49].

Recurrent inversion is characterized by a large inversion of a portion of chromosomes (Figure 1). It is estimated that an average of 11.6 Mbps is flipped in a normal genome [50]. Recurrent inversions are flanked by retrotransposons or by segmental duplications that often exceed the length of sequencing reads or library inserts. This technical challenge caused this genetic variation class, which might represent more than 0.6% of the genome, to be underexplored [50]. Recurrent inversions exhibit a sex-chromosomal bias and co-localize with critical regions of genomic disorder. 

Another particularity about the importance of CIN in germline cells and in structural variance in humans is pediatric cancer. Pediatric tumors show a higher frequency of germline alterations, copy number alterations, and structural alterations such as enhancer hijacking events and gene fusions as possible oncogenic drivers and chromoplexy [51,52]. A study from 33 cancer types has shown that 8% of 10,389 analyzed cases presented pathogenic variants such as CNV and large deletions in tumor suppressors, including ATM, BRCA1, and NF1 [53].

## 5. Chromosomal Instability in Cancer

In the past decade, thousands of patient tumors have been sequenced and have revealed that cancer genomes are highly complex and heterogeneous [54]. Sequencing tumors and metastatic sites from the same patients has allowed the drawing of the tumors’ phylogenetic tree and the determination of early and late events [15,55,56,57,58,59]. Something very intriguing is the similarity and parallelism between the genomic complexity history of tumors and genome evolutionary changes. Drastic events such as chromosome rearrangements and gain/loss of chromosomes both draw the evolution of them to adapt and progress to new environments and pressure. Whole genome doubling in tumorigenesis [15,55,56,57,58] is also an early event supporting the creation of genome diversity as observed early before the diversification leading to vertebrates (Figure 3) or as observed in plants [44]. Interestingly the duplication of their genome may occur multiple times during tumor development, leading to highly complex cancer genomes [15,60].

### 5.1. Tumor Clonal Heterogeneity

Tumors arise from one cell that will generate a plethora of diverse clones with independent genomic alterations (Figure 1). Tumorigenesis involves modification in the genome, and mutations are key in the process. However, chromosomal instability is indispensable for generations of diversity and is one of the hallmarks of cancer [61]. Tumor heterogeneity consists of intra-tumor (within a tumor) and inter-tumor (tumor by tumor as, for example, metastatic lesions versus primary site) heterogeneity. Several sub-clones can derive from the same clones. The use of single-cell analysis tools has allowed the understanding of the genomic complexity of clonal and sub-clonal diversity, as well as their dynamic and clonal selection [62,63,64]. Importantly, the clonal selection during therapy or metastatic progression leads to a temporal restriction of diversity in a bottlenecking process (Figure 2). However, invasion is also multiclonal, as cells from Breast Ductal carcinoma show one or more clones escaping the ducts and migrating into the adjacent tissues to establish the invasive carcinomas [65].

### 5.2. Different Chromosomal Changes Observed in Tumor Evolution

Eighty to ninety-seven percent of all tumors present detectable CIN [12,60], supporting contact evolution. Besides the classical and simple genomic aberrations such as mutations, deletions, translocations, and insertions, cancer cells can present complex chromosomal rearrangements like chromothripsis and chromoplexy [18]. Chromothripsis is a chromosomal instability phenomenon, a genomic catastrophe, where hundreds of chromosomal rearrangements occur during one single event in a localized region of one or few chromosomes. This type of chromosomal rearrangement is highly frequent in cancer, with a prevalence of 49% to up to 80% [66]. Chromothripsis is associated with the formation of circular extrachromosomal DNA (ecDNA) [67,68] as well as with segmental deletion. Chromothripsis increased with whole-genome duplications in most cancer types [69]. A new study claims that those drastic changes arose from mitotic catastrophes that rapidly shape new genetically distinct clones. The analysis of these phylogenetic trees revealed that complex structural events, including chromothripsis, are major drivers [66,70]. As performed for mutations [71], different chromosomal instability patterns are observed in cancer upon the origin of insults and can lead to a specific signature [12,60]. Some signatures predict drug response and identify new drug targets [12]. 

### 5.3. Punctuated Burst and Genomic Evolution

The Darwinian model or gradualism of clonal selection, where a subset of genetic lesions drives tumor evolution and progression in a stepwise manner, is widely accepted as the mode of evolution of malignant cells under therapy or basal conditions [72,73,74,75,76]. However, recent findings in prostate, pancreatic, and triple-negative breast cancer (TNBC) call into doubt this paradigm, raising the question of whether gradualism is the only mode of evolution. Such new studies highlight events of punctuated bursts, followed by the stable clonal expansion that forms the tumor mass [18,55,77,78]. Episodes of transient CIN can explain the genomic burst of diversity, leading to the creation of structural or numeral chaos leading to a two-phased pattern of evolution [79,80].

### 5.4. CIN and Tumor Progression

Chromosomal instability is primordial for tumor progression and supports tumor progression and metastasis. In fact, tumors harboring high levels of CNA have a worse prognosis than high mutation rate tumors because of their ability to escape the immune system [26]. Chromosomal instability is even enriched in metastases of several cancer types [81,82]. Chromosomal instability accelerates the evolution of resistance to anti-cancer therapies [83]. Tumor aneuploidy predicts survival following immunotherapy across multiple cancers, and an elevated aneuploidy score is an independent and complementary predictor of overall survival for patients with low tumor mutational burden tumors [84]. Furthermore, CIN also appears to be indispensable for the success of metastasis. Tumor cells with high CIN levels activate the pro-inflammatory pathway cGAS-STING to support their survival and growth [81].

## 6. Possible Origins of Chromosomal Instability and Its Study

There are several potential causes of CIN, including genetic mutations, errors in cell division, and exposure to certain environmental factors. Hereinafter, we delve into some of them.

### 6.1. Mitotic-Cell Cycle Errors

A mitosis is a delicate event that must be executed with high fidelity to ensure genomic integrity. However, errors in cell division can contribute to the development of malignant karyotypes in cancer [85]. This can be due to a variety of factors, including defects in the spindle assembly checkpoint (SAC), cohesion defects, problems with kinetochore attachment, or an increase in the number of centrosomes [85].

Issues during mitosis can conclude with gross gain or loss of chromosomes, such as during mis-segregation, where a full chromosome goes to the wrong daughter cell. Importantly, lagging chromosomes during mitosis can produce the formation of micronuclei. In fact, if a chromosome is late during the nuclear envelope (NE) formation and is not clustered with the other chromosomes, a proper NE will form around it, forming a separate small nucleus close to the main nucleus, called micronuclei (MN). Micronuclei can contain a full chromosome or a chromosome fragment [86]. MN are, in fact, a major source of DNA damage and chromosomal instability due to NE rupture [87]. Furthermore, MN can be cleared via autophagy, participating in the creation of chromosomal unbalance [88].

### 6.2. Transient Nuclear Envelope Rupture (NER)

Recent work has demonstrated the existence of transient Nuclear Envelope Rupture (NER) during interphase as origin of several form of chromosomal instability. The NE under certain stress can collapse, exposing the genome to the harmful cytoplasm for several minutes to the main nucleus or even permanently in the case of micronuclei [86]. Such unexpected events have drastic consequences on genome evolution as NER can lead to complex and simple chromosomic events such as chromothripsis, extended above [68,89,90,91,92,93,94]. 

NER happens in micronuclei, where the envelope is fragile and tends to disrupt without the possibility of proper repair [87,95]. In another case, NER happens in cells with chromatin bridge, when cells experiencing telomere fusion connect two daughter cells. This implies the generation of additional tension forces affecting the NE during movement, leading to NER that can last up to two minutes [94]. Cells during migration can also experience NER of their nuclei [96,97,98,99]. Interestingly, immune attacks can fail and can contribute to mutagenesis. Cytotoxic T lymphocytes often fail to kill target cells during a one-one-to-one conjugation and can lead to transient NER, leading to DNA damage [100]. Such a “failed” attack then participates in the generation of genomic diversity and progression toward resistance.

The mechanisms responsible for the genomic aberrations linked with NER are still under debate. Some authors have demonstrated that in the cytoplasm, the unprotected DNA can be attacked by DNAses such as TREX1 [101] using the immune DNA mutator APOBEC [92] or can break due to mechanical forces during migration [91]. Furthermore, the loss of compartmentalization can affect the replication inside the micronuclei, provoking a desynchronization with the main nucleus. Thus, the main nucleus may start the mitosis too early for the DNA trapped inside the micronuclei, which is not folded nor protected and can lead to its pulverization within the cytoplasm, resulting in chromothripsis [89,91]. Another explanation is that micronuclei accumulate large amounts of RNA–DNA hybrids that can lead to DNA fragmentation due to pathological DNA-base excision repair [102].

### 6.3. Double-Stranded Breaks

Double-stranded breaks (DSBs) are a type of DNA damage that occurs when both strands of the DNA helix are broken—a chromosome break. They can be caused by several factors, such as exposure to ionizing radiation, reactive oxygen species, and certain chemical agents or by the activity of endogenous enzymes like topoisomerases, helicases, and primases. DSBs are a common type of DNA damage and are considered the most dangerous, as they can lead to chromosomal aberrations, genomic instability, and cell death if not repaired properly. The repair of DSBs is a complex process, and it can be accomplished mainly with non-homologous end joining (NHEJ) or homologous recombination (HR) pathways. The choice of repair pathway depends on the cell cycle stage, the type of DNA damage, and the cell type. Some of the genetic causes of CIN associated with DSBs include mutations in genes that are involved in maintaining the integrity of chromosomes, such as the *BRCA1* and *BRCA2* genes, which are involved in the HR pathway and are associated with an increased risk of breast and ovarian cancer. Other genetic causes of CIN include mutations in genes that act as sensors of DNA damage, such as the *ATM, ATR,* and *DNA-PK* genes. But in case of repair failure, cells risk having a broken chromosome that can lead to the formation of micronuclei. In fact, a recent study using CRISPR-Cas9 gene editing has shown that a single DSB can lead to a cascade of events resulting in the formation of micronuclei and chromosome bridges [103]. Such simple DSBs can be amplified into far more extensive genomic alterations during subsequent mitosis, leading to a myriad of genomic diversity.

### 6.4. Replication Stress

Evidence suggests that impaired replication fork progression and increased DNA replication stress lead to CIN [104]. Replication stress can be induced by several factors, including oncogenes [105], low nucleotide concentrations [106], and challenging DNA structures [107,108], and is responsible for CIN in multiple cancer types [109,110]. Stalled forks caused by replication stress can trigger different responses. Normally, high levels of replication stress lead to the activation of ATR and Chk1 checkpoints and senescence, acting as an obstacle to tumor initiation. However, low levels of replication stress can bypass these sensors and continue with cell proliferation [111], even promoting unusual mitotic DNA synthesis (MiDAS) to promote replication in these lagging regions before the completion of mitosis [108,112]. Failure to activate these pathways may result in DNA breaks, which can cause mitotic errors in the following cell division and lead to chromosomal abnormalities, including lagging chromatin, anaphase bridges, aneuploidy, and micronuclei formation [109,113].

DNA copy number alterations (CNAs) caused by replication stress can be detected through cytogenetic analysis, which identifies DNA breaks in genomic regions called common fragile sites (CFS), or by using various approaches to map regions that undergo late mitotic replication, such as array comparative genomic hybridization (aCGH) [114,115], ChIP-Seq of FANCD2 following replication stress [116], or MiDAS-Seq [117]. Although these methods offer an excellent positional resolution, their ability to detect the broad spectrum of possible CNAs is restricted to those that survive long-term clonal outgrowth or occur frequently enough to be detected in a population analysis [114,115]. This limitation does not permit the analysis of intratumor heterogeneity, which is important to understand tumor development and skew our knowledge of cancer genome evolution.

To understand the precise and acute changes to the genome upon replication stress leading to distinctive CNA landscapes, single-cell analysis, as low-pass single-cell whole genome sequencing of G1 cells after replication stress induction [104,118,119], providing insights into the molecular mechanisms that fuel chromosomal instability in cancer. Therefore, understanding the exact mechanisms and consequences by which replication stress leads to genomic alterations and cancer evolution is an important but not fully understood task.

### 6.5. Implication of Viral Infection

Several viruses need the integration of DNA viral sequences into the host genome for their amplification to have mutational consequences. However, viruses are also implicated in several chromosomal reorganizations, and recent advances in DNA sequencing technologies support the characterization of specific patterns of structural variation resulting from viral infection.

Five percent of all cancers are caused by human papillomavirus (HPV), which is responsible for 95% of cervical cancers and is also involved in some head and neck cancers. HPV infection concludes in the integration of viral sequences into the host genome. HPV infection is then associated with mutations often localized into integrated hotspots but is also involved in the generation of CIN. Whole-genome sequencing revealed HPV integrants flanking and bridging extensive host genomic amplifications and rearrangements, including deletions, inversions, and chromosomal translocations [120]. Other viruses, such as HIV, are also associated with the formation of micronuclei and can lead to the formation of chromosomal anomalies [121]. Hepatitis B Virus (HVB) infection has been associated with chromosomal instability in cancerous and non-cancerous liver genomes and HBV–DNA integration is known to be the cause of chromosomal rearrangements in human hepatocellular carcinoma. Recent ‘long read’ sequencing analysis has revealed that HBV–DNA integration mediates interchromosomal genomic rearrangements that lead to megabase-size telomeric deletions [122]. As such, a specific virus-driven pattern exists and needs further analysis, which is now possible thanks to new sequencing strategies. Furthermore, there is currently speculation that chromothripsis might be driven by viruses such as γ-herpes viruses [123]. 

### 6.6. Expression of Meiotic-Specific Proteins 

Meiotic-specific proteins are a group of proteins that are specifically expressed and play important roles during the meiotic stage of the cell cycle. However, in some cases, meiotic-specific proteins can be expressed during mitosis as a result of genetic mutations, chromosomal abnormalities, or other disturbances in the cell cycle. This can lead to chromosomal instability, micronuclei formation, cell death, or even cancer. These events can occur in normal somatic cells or in cancer cells, the expression of meiotic proteins during mitosis has been associated with chromosomal instability, DNA damage, and aneuploidy, which are hallmarks of cancer [124,125,126,127,128]. Importantly, a study has revealed that overexpression of the meiotic cohesin Rec8 in mitotic fission yeast cells leads to the uniparental disomy of chromosomes and CIN in this organism [129]. Another meiotic gene is PRDM9, described below in more detail. A study made with 1879 cancer samples across 39 distinct cancer types discovered that PRDM9 was unexpectedly present in 20% of these tumors, even after applying strict gene homology adjustments [130].

### 6.7. P53 and Other Factors Linked to Tumors 

Cancer cells have a tendency to accumulate a variety of mechanisms that result in genomic instability. This instability allows cancer cells to evolve and accumulate errors, which enables them to survive and progress to increase their fitness. One of the most commonly mutated genes in cancer is *TP53*, also known as the “guardian of the genome” [69]. When *TP53* is mutated or deleted, cells lose their ability to correct errors, leading to genomic diversity and allowing the cells to survive. For example, in mouse models, *TP53* LOH leads to the accumulation of deletions, genome doubling, and the emergence of gains and amplification [131]. 

Aneuploidy can lead to the activation of p53 [132], but how this activation is triggered has been a controversial matter, with proposed mechanisms including activation via p38 stress kinase [133] or elevated levels of DNA damage from reactive oxygen species [134]. Another theory proposes that chromosome segregation errors produce DNA breaks that activate p53 through the activation of ATM [135]. A study by Soto et al. has investigated the direct consequences of de novo aneuploidy on p53 activation. Interestingly, the authors found that p53 was not always activated by aneuploidy *per se*, as not all whole-chromosome aneuploidies triggered p53 activation, and at least a subset of these is tolerated in p53-proficient backgrounds [119].

Interestingly, studies using Next-Generation Sequencing to analyze human tumors have associated TP53 (encoding human p53) mutations with genomic instability features like CNA [136], chromothripsis [137], whole-genome doubling [138], and also being involved in displaying intratumoral heterogeneity [139], but how mutant genomes emerge, and influence tumorigenesis remain poorly understood. A study conducted using a mouse model of pancreatic ductal adenocarcinoma found that sporadic loss of heterozygosity in Trp53 (the mouse p53 gene) occurs before cancer onset and leads to the acquisition of malignant properties facilitated by p53 inactivation. This process involves four distinct phases of genome evolution that occur sequentially, which include Trp53 loss of heterozygosity, accumulation of deletions, genome doubling, and the emergence of gains and amplifications. Each of these phases was associated with specific histological stages that occur across the premalignant and malignant spectrum [131].

Other mechanisms specific to tumorigenesis are also implicated in CIN. Recently the expression of APOBEC3A, a cytidine deaminase upregulated across cancer types, was shown to drive chromosomal instability and promote pancreatic cancer metastasis [140]. The epigenetic program Epithelial to Mesenchymal Transition (EMT) is also involved in transient CIN [97].

### 6.8. Study of CIN in Genome Evolution of Cancer

To study CIN in the genome evolution of human cancer, there are several practices that researchers can implement, including:

- Whole-genome sequencing: Whole-genome sequencing provides a comprehensive view of the entire genome, including chromosomal rearrangements and copy number alterations that result from chromosomal instability.
- Comparative genomic hybridization (CGH): CGH is a technique that compares the copy number of genomic regions between a reference genome and a test genome. This technique can identify regions of gain or loss that result from chromosomal instability.
- Fluorescence in situ hybridization (FISH): FISH is a technique that uses fluorescent probes to bind to specific genomic regions. This technique can be used to visualize chromosomal rearrangements and copy number alterations.
- Single-cell sequencing: Single-cell sequencing can provide insight into the genomic heterogeneity within a tumor and can reveal subpopulations of cells with different patterns of chromosomal instability.
- Cell culture and xenograft models: Cell culture and xenograft models can be used to study the effects of CIN on tumor growth and response to treatment.
- Bioinformatic analysis: Bioinformatic tools can be used to analyze genomic data and identify patterns of chromosomal instability, including chromosomal breakpoints, copy number alterations, and structural variants.

By using these techniques and tools, researchers can gain a better understanding of the mechanisms driving chromosomal instability in cancer and how it contributes to the evolution of tumors. This information can ultimately lead to the development of new therapeutic strategies that target chromosomal instability and prevent the evolution of drug-resistant tumors.

## 7. Germ Cells and Meiotic Program as Drivers for Genome Evolution

Around 50–70% of human cleavage embryos are aneuploid, highlighting the high levels of CIN during the generation of offspring [141,142]. Most aneuploid embryos do not develop to term, making aneuploidy in embryos a leading cause of miscarriages and infertility. Errors during meiosis explain a great portion of aneuploidy and CIN in early embryos. We will then develop here in detail the different steps during meiosis to understand the origin of CIN and the generation of chromosomal diversity. However, aneuploidy is also thought to arise during the mitotic divisions of the embryo during early development. A recent study [22] shows that improper clustering of parental genomes with nucleoli in each pronucleus during fecundation can lead to lagging chromosomes and the formation of micronuclei.

**Figure 4:**
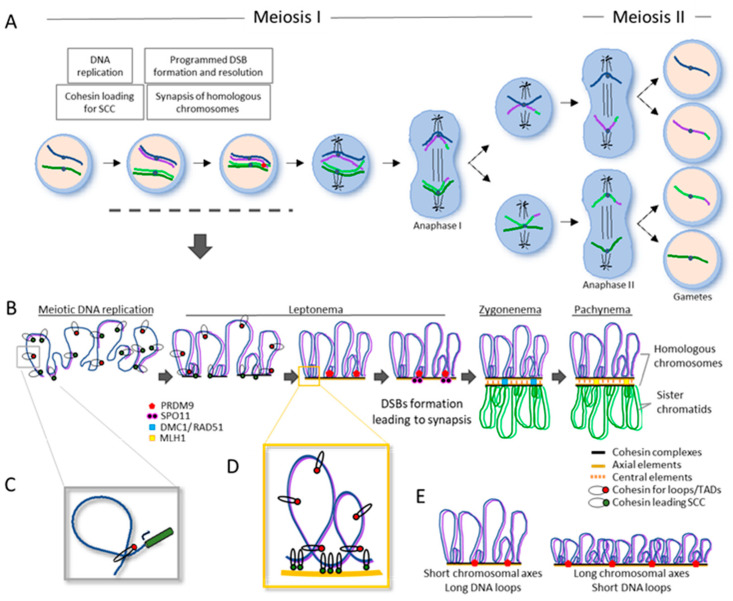
Meiosis, key processes, and factors involved. (**A**) Different phases and key processes for Meiosis I and Meiosis II, from meiotic replication to the formation of haploid gametes. The use of only one pair of homologous chromosomes (blue and green) and their replication to sister chromatids (purple and light green) have been used for simplification. In Anaphase I, homologous chromosomes segregate, and in Anaphase II, sisters are pulled apart. Key processes during Prophase I (dashed line) are detailed in B, where different phases are represented. (**B**) Along meiotic DNA replication, cohesin complexes are loaded, leading to SCC (sister chromatid cohesion). From leptonema to pachytene, sister chromatids begin to condense along a proteinaceous structure formed by cohesin complexes, such as REC8, and axial elements, such as HORMAD1. Meiotic chromosomes are organized as DNA loops anchored to the axial elements, where proteins involved in the formation of DSBs, such as PRDM9 and SPO11, are recruited. These breaks are repaired where its resection leading to 3’ ends is key, covered by RPA and then by the recombinases RAD51 and DMC1 at zygonema. These two proteins catalyze strand invasion and will help with the homologous chromosomes’ recognition. In the meantime, that homologous recombination takes place, synapsis of homologous chromosomes occurs, with the polymerization of the central elements SYPs between both axials, leading to the Synaptonemal Complex (SC) formation. Using pachynema, SC is completed, and recombination intermediates can be resolved either as COs or non-COs. The resolution of COs is mediated by MLH1 proteins that will ultimately lead to chiasma formation. (**C**) cohesin occupancy mediates a role in transcription regulation by bringing two DNA regions together. (**D**) Schematic representation for distinct chromosomal localization pattern for cohesin mediating: SCC between sisters, loops and TADs formation, or transcription regulation. (**E**) The length of chromosomal axes and the size of DNA loops are inversely related, resulting in different numbers of DSBs formed by the protein SPO11. Figure adapted from [145].

Meiosis is a specific cell division that only takes place in the germ line of organisms with sexual reproduction. The germ line is composed of pluripotent and mitotic self-renewing stem cells, which finally trigger the meiotic program to sustain gamete production, both oogenesis (egg production) and spermatogenesis (sperm production) (Figure 4A). Meiosis comprises two consecutive rounds of cell divisions, meiosis I and meiosis II, with the peculiarity that only a single round of S-phase genome replication takes place in meiosis I and not during meiosis II. As a result, four haploid daughter cells form, each containing only one set of chromosomes (half the number present in the original cell) (Figure 4A). After fertilization, the whole set of chromosomes that define the species is recovered [143,144].

Therefore, unlike the rest of the somatic tissues, germ cells are the only ones that contribute genetically to descent, and then the information contained is passed from generation to generation. In this regard, the protection of genome information of this tissue is essential for the perpetuation of species. Then, it is possible that germ cells possess unique strategies to protect genetic information. In addition, the meiotic program is highly regulated as defects are linked with infertility, miscarriages, and congenital diseases. However, at the same time, meiosis is linked with a generation of genome diversity with the final goal of breeding diversity in the offspring for an adaptative response to the environment and increasing the fitness of the species. The first level of genetic shuffling and generation of new allele combinations comes from the reduction in chromosome number and a random assortment of them during meiosis and the fertilization of gametes after sexual reproduction from different parents. A second level implies a molecular mechanism that involves programmed break points along the DNA, repairing with and linking homologous chromosomes forming the crossovers, essential for proper chromosome segregation at meiosis I. As a consequence, an exchange of material takes place, generating a new combination of genetic information.

Therefore, germ cells contain the genetic information that reflects the evolutionary history and holds the potential for future developments of species. Understanding how the genome is organized in germ cells, the maintaining of genome stability along its self-renewing during the meiotic program, and during chromosome segregation is fundamental to understanding fertility and its impact on genetic diversity and the evolution of species and might highlight similar mechanisms in cancer. 

### 7.1. Chromosomal Axis and Synapsis in Genome Stability and Evolution 

Two rounds of cell divisions, meiosis I and meiosis II, are required to produce gametes. Before entering meiosis, germ cells are typically diploid and possess two copies of each chromosome, called homologous chromosomes. A single round of DNA replication results in four copies, each consisting of two homologous chromosomes made up of two sister chromatids. The sister chromatids of each homolog are attached during replication with ring-shaped protein complexes called cohesins. During meiosis I, the homologous chromosomes segregate, while during meiosis II, in a similar way to mitosis, sister chromatids segregate, resulting in four products with half the genetic information (Figure 4A,B) [146].

The special segregation of homologous chromosomes during Meiosis I is achieved through a mechanism involving both synapsis, the pairing of two homologous chromosomes, and DNA recombination between them (Figure 4B). These two relevant events take place during prophase I, maybe the most important phase of meiosis, which lasts the longest. Prophase I is broken down into four stages called leptonema, zygonema, pachynema, and diplonema, according to these events and the associated dynamics of meiotic chromosomes, as there are dramatic chromosomal movements and chromatin remodeling during this phase [146]. In short, the joining of homologous chromosomes begins during leptonema with DNA organization into loops anchored to a chromosomal axis formed by a protein scaffold structure along the chromosomes, made of cohesin (REC8 or RAD21L in mammals) and specific proteins (the HORMA domain-containing proteins) constituting the axial element of the synaptonemal complex (SC) along each homologous chromosome [147,148,149,150] (Figure 4B). After specific homologous recognition takes place, the synapsis or polymerization of central elements (SYCP proteins) between both axes occurs [151,152]. Simultaneously during leptonema, meiotic recombination starts with the creation of DSBs (double-stranded breaks) by the SPO11 endonuclease protein [143]. During zygonema, DSBs are repaired, resulting in synapsis and the exchange of genetic material between homologous chromosomes, leading to crossovers (COs) (Figure 4B) [153]. Along the next phase, pachynema, chromosomes finish synapsis completely, forming bivalent structures, recombination is completed, and COs are resolved. During diplonema, the chromosomes begin to condense and become more compact in preparation for cell division. In this phase, the SC and cohesin begin to release, leading to the chiasmata, the visual and physical manifestation of attached homologous chromosomes, where the recombination occurs. This allows the accurate segregation of homologous chromosomes during anaphase I. Later on, gametes are obtained with the completion of cohesin release from the arms of the sister chromatids during anaphase II. 

Defects in these events can result in nondisjunction, producing aneuploid gametes with abnormal numbers of chromosomes, which leads to aneuploidy and sterility [154]. Overall, errors in gametogenesis leading to aneuploidy are a relatively common occurrence in humans, with estimates suggesting that it occurs in 10–30% of cases, particularly increasing sharply with maternal age [154]. 

However, alteration of normal ploidy can have beneficial effects, such as accelerating genetic diversification and rapid adaptation. Studies done on unicellular organisms have shown that rapid adaptation to stress environments is often characterized by aneuploid states, which are also associated with changings in gene expression and an increase in mutational rates and accumulation of them to increase fitness [155,156,157]. Accordingly, a study in the plant Arabidopsis has shown that the REC8-cohesin complex is linked to multigenic adaptive evolution in autopolyploid and whole genome duplication [158]. Robertsonian (Rb) chromosome fusions, expanded upon at a later point (see Section 7.3.4), are another example. Further to their impact on fertility, their consequent secondary defect in synapsis leads to chromosomal incompatibilities between divergent lineages when meiotic contacts during prophase I through the SYCP3 filament were studied. This contributes to chromosomal speciation, as it has been described in the rodent Alai mole vole (*Ellobius alaicus*) in the family Cricetidae [159].

### 7.2. Impact of Three-Dimensional Chromatin Structure on the Control of Gene Expression

During gametogenesis, further to the Synaptonemal Complex (SC) configuration between homologous chromosomes, a genome undergoes a 3D reorganization, and insulator proteins such as CTFC and cohesin complexes play a role in organizing chromatin into compartments (open/closed chromatin), topologically associated domains (TADs), and DNA loops. This organization ultimately affects both the physical arrangement of chromatin and the regulation of gene activity [160,161,162] (Figure 4C,D). This reveals that the spatial conformation of genomes is given by a close interplay between gene function and chromatin organization. Research in mice has shown that the chromatin architecture is reprogrammed during the development of germ cells [163]. Therefore, it is not surprising that recent studies have shown that functional plasticity under a higher-order genomic organization is passed on to offspring, ultimately contributing to the formation of new allelic variants on which natural selection and evolution can act [162]. 

Importantly, it is known that the repair of DSBs occurs in the context of this axial chromosomal structure [164,165]. A relatively low level of compartmentalization during prophase I has been linked to the specific events that occur during this phase, such as chromosomal movements, chromatin condensation, and the formation and repair of DSBs. Later on, during gametogenesis, there is a close interplay between increasing compartmentalization and differential gene expression. However, recent discoveries have improved our understanding of the link between the dynamics of chromatin organization and its role in mouse spermatogenesis beyond gene expression. Briefly, chromatin conformation plays a crucial role in the generation and resolution of DSBs, which have significant implications for evolution, as discussed above [162]. 

In this sense, large-scale chromosomal changes can also result in inherited diseases, genome instability, and cancer by altering the expression of genes in those reorganized genomic regions. Along with this view, it has been reported that disorders of domain architecture and chromatin organization caused by inversions, fusions, or insertions can lead to the activation of oncogenes or the emergence of new gene functions [166,167,168]. Hence, similar underlying mechanisms are responsible for both evolutionary changes in the genome and genetic instability diseases.

### 7.3. DNA Recombination and Repair as a Mayor Source for Evolution

DNA recombination is a central process during meiosis that leads to a physical attachment between homologous chromosomes. This step is called Crossing-over (CO) and is a universal feature that is necessary for faithful chromosome segregation during meiosis and sexual reproduction, maintaining the genome integrity of species. Additionally, meiotic recombination shuffles alleles along homologous chromosomes, leading to genetic diversity in sexual gametes and offspring [169]. In this sense, understanding the mechanisms and factors involved in meiotic CO formation and resolution can provide insight into biodiversity, but if not well-regulated, it can lead to repair errors resulting in sterility or miscarriages. On the other hand, proper regulation of new meiotic CO formation and resolution could be adaptive, supporting the evolution of species or the emergence of new ones.

#### 7.3.1. Recombinational Hotspots as a Factor for Genome Instability and Evolution

DNA recombination begins with programmed DSBs mediated by an endonuclease called SPO11. This protein is highly conserved across eukaryotic evolution [143,165]. Along with SPO11, other proteins are involved in the control and formation of DSBs, such as PRDM9, MEI4, REC114, or HORMAD1 in mice [164,165,170]. Due to its importance, this process is highly regulated throughout the meiotic cell cycle, particularly at leptotene and prophase I. Breaks are concentrated at the called recombination hotspots, where SPO11 and other factors are involved in the formation and repair of DNA breaks in the early stages of meiosis, and it is highly regulated, including the total number and genome distribution of DNA breaks [6]. Recombination hotspots have been found in localized short regions of hundreds to thousands of base pairs in many species, including yeast, plants, and all vertebrates studied to date [171]. However, in some species, such as nematodes, the recombination landscape appears more uniform and lacks these hotspots [172]. There are between 150 meiotic DSBs per cell in humans and around 200–300 in mice [173,174]. It is known that human hotspots are normally 1–2 kb in size, spaced 50–100 kb apart, accounting for no more than 20% of the genome, and only ~5% to 30% (depending on the organism) go on to produce COs [175]. Centromeres are normally recombination deserts, whereas, in (sub)telomeres, recombination rates increase. Mirroring what has been described in mice [176], hotspots are localized in genic and intergenic regions likewise. This suppression of recombination around centromeres has been described as not related to the presence of the heterochromatic domains, which are typically associated with satellite DNA in this area, at least in horse spermatocytes [177]. Some studies also unveil the importance of specific DNA motifs and epigenetic modifications in determining recombination hotspots [178,179]. 

#### 7.3.2. Factors and Complexes Define Recombinational Hotspots

##### Cohesin Complexes and Synaptonemal Complex Axial Elements 

As previously mentioned, the way genomes are packaged is likely another factor influencing species-specific differences in recombination rates. The mean number of DSBs per cell is influenced by DNA compaction through SC length and DNA loop size (Figure 4E). There is a close interplay between SC assembly, the organization of DNA loops, and the formation of DSBs [144,180]. It has been noted that chromosomes with longer DNA loops and shorter SC axes showed a reduced number of DSBs in early prophase I. An explanation could be that shorter SC axes could anchor fewer DNA loops but longer, providing less scaffolding for the formation of DSBs and recombination rates. Likewise, loop sizes were inversely correlated with CO density [6,144,181] (Figure 4E).

Recent studies have highlighted the importance of certain factors in shaping genomic architecture and, subsequently, evolution. Cohesin-mediated DNA loops are organized along the SC axial scaffold, anchoring as long DNA loops and establishing a physical first level that defines hotspots [151,182] (Figure 4D). Cohesin complexes, HORMADs, and SC proteins correlate with these recombination hotspots, as reflected by the interactions observed at shorter distances (2.5–4.5 Mbp). In this sense, a recent paper found that a majority of the cohesin complex during spermatogenesis is localized within promoter regions of genes located in DNA loops away from the axes [162] (Figure 4C). In this regard, meiotic-specific cohesin provides an integrated structural and functional framework for the 3D organization in germ cells and spermatocytes in mice, manifesting a fine-tuned balance among recombination, chromatin remodeling, architectural proteins, and gene expression during spermatogenesis [162].

##### Protein PRDM9

Among species with recombination hotspots, locations are specified by PRDM9 (PR domain 9) binding. PRDM9 is a specific early meiosis protein expressed only in testes and ovaries [130]. This protein presents a zinc finger (ZnF) motif that recognizes specific DNA sequences in the genome and adds H3K4me3 and H3K36me3 marks at nearby nucleosomes close to DSBs in early meiosis, recruiting the DSB repair machinery and SPO-11 in early stages of meiosis and determining recombination hotspots [183,184]. In terms of evolution, the PRDM9-ZnF motif recognizes a high variability of DNA motifs, which modification can be translated into a redistribution of recombination hotspots [183]. In this sense, different distributions of DSBs have been described in humans with different PRDM9 alleles [185] and in mice [186], providing new clues on a factor that generates new DSB sites, which could be a source of genetic instability and indirectly have important consequences in evolution. PRDM9 is not found in all vertebrates; it is absent in certain birds, canids, and fishes. However, orthologs of PRDM9 have been identified in some of these groups [8]. Interestingly, mammals with PRDM9 have the fastest-evolving zinc fingers (ZF) array [175,187]. Then, these mammals generate novel sets of hotspots leading to rapid turnover in the recombination landscape between populations and species and thus have a role in the evolution rate. The selection pressure to explain the fast evolution of the PRDM9 ZF remains unclear. It could be explained as an assurance of recombination hotspots and to avoid inefficient DSB repair leading to reduced fertility or sterility [186,188].

On the other hand, in species without PRDM9, the rapid evolution of recombination hotspots is not seen. For example, in species of birds lacking an ortholog of PRDM9, the locations of recombination hotspots are conserved over long evolutionary time scales, reducing evolution over time [8]. Species that do not use PRDM9 and have more stable recombination locations tend to direct recombination in promoter-like features, as opposed to species that use PRDM9 and experience rapid turnover of hotspot landscapes, which tend to recombine away from promoters and coding-protein sequences, even in some subfamilies of transposable elements in mammals using ZF domain recognition [189]. These different dynamics have important consequences for genome structure and, therefore, evolution [190,191]. As expected, recombination hotspots have higher rates of point mutations, insertions, deletions, and translocations. Therefore, directing recombination to non-coding regions might be an advantage in avoiding genome instability. In this regard, recombination at transcription-related loci could uncouple coding regions and their regulatory elements, leading to transcriptional regulation of genes or negative epistasis [192].

##### Chromosome Organization as a Modulator of Recombinant Landscape

The normal progression of the meiotic cell cycle involves reshuffling of the genome, which is mediated by programmed DSBs and provides diversity among organisms with new combinations of alleles on which natural selection can act. However, sometimes large-scale genomic changes, such as inversions, translocations, fusions, and fissions, occur, leading to infertility or providing new sources of variation on which natural selection can act and pushing speciation. Several genetic and mechanistic factors, such as PRDM9 in mammals [184], affect the recombination landscape and the combinatorial effect of chromosomal rearrangements, leading to speciation.

On the other hand, chromosomal rearrangements can play an important role in further modifying both the structure and regulation of genes located near the affected regions, as proposed by the “suppressed recombination” model [193]. This is because recombination is suppressed within rearranged segments, inversions, or chromosomal fusions, resulting in a reduction of COs within these regions [194]. As a result, there is a reduction in gene flow around reorganized genomic regions, and chromosomal reorganization could lead to a combination of alleles, polymorphisms, or haplotypes that provide a certain degree of selective advantage or accumulate genetic incompatibilities, ultimately contributing to species evolution and divergence in the long term. As a result of chromosome reorganizations, there is an alteration of the recombination maps, leading to genetic differentiation across genomes, referred to as “genomic islands of divergence” [195,196]. Evidence for this has been described in mammals, such as between human and macaque lineages, where recombination patterns have undergone rearrangements that could be favoring adaptive alleles in the immune response [197]. Other studies have described similar reductions in recombination landscapes in mice due to Robertsonian translocations, also related to alterations in epigenetic signatures for heterochromatinization [193,198].

Future comparative research on recombination hotspot landscapes, speciation genes, and different chromosomal rearrangements and how these changes in chromosome structures affect recombination should be conducted to enhance our understanding of the main pathways for genome reshuffling and evolution.

#### 7.3.3. Pathways of Repair of Meiotic DSB to Chiasma Formation 

Not all breaks mediated by SPO11 are resolved by CO formation and subsequent chiasma formation, point of contact, and the physical link, between homologs. DSBs lead to an orchestrated DNA damage response mediated by the proteins ATM (ataxia telangiectasia mutated) and ATR (ATM-Rad3-related) due to the phosphorylation of histone H2AX on serine 139 (γH2AX) [199]. After several coordinated steps involving DNA resection, which leads to 3’ strand overhangs where monomers of RPA are located and displaced by the recombinase RAD51 and/or DMC1, forming nucleoprotein filaments that catalyze strand invasion. Different repair pathways can mediate the repair of the processed breaks with two possible final outcomes: COs leading to the chiasmata connecting both homologous chromosomes or non-COs (NCOs) generating gene conversion without a connection between chromosomes. To assure homeostasis and correct chromosome segregation during the first meiotic division, CO assurance in mammals is carefully controlled, and the total number is relatively constant among cells [174]. Along with this view, several studies in different organisms have revealed that the number of meiotic DSBs exceeds the number of chiasmata, indicating that the majority of DSBs are resolved as NCOs [174,200]. A highly regulated genetic control determines the fate and distribution of COs. This is known as CO interference, which reflects the restriction on a new CO taking place near another established CO. This restriction decreases with chromosomal length, leading to a spaced distribution across chromosome axes [144,180]. All of these aspects, from meiotic pre-replication to DSB formation to a controlled resolution until COs, are relevant for understanding the diversity of genomes at the individual level but also the genetic variation leading to evolutionary change.

#### 7.3.4. Defects in Meiotic Recombination Leads to Chromosome Rearrangements 

While it has been reported that disruptions of chromosome architecture such as inversions, fusions, or translocations are associated with genetic instability and cancer due to oncogene activation and novel gene functions, there is a parallelism in organism evolution due to intra- or interchromosomal alterations in the germ line or during the meiosis program, which can alter normal segregation patterns and contribute to evolution [8,165]. Otherwise, failures in the meiotic program can also lead to aneuploidy, infertility, genetic abnormalities, and congenital disease [201,202].

##### Large Chromosomal Rearrangements as Drivers for Speciation

One chromosomal rearrangement that affects the recombination landscape and is linked to miscarriages, infertility, or aneuploid descendance in humans and pushes evolution is the Robertsonian (Rb) fusions or translocations, the most common balanced chromosomal rearrangement in nature. Rb translocations typically involve two acrocentric chromosomes, homologous or non-homologous, and result in the fusion of both long arms to form a single metacentric chromosome, plus the loss of both short arms. Aneuploidy, and loss of genetic information are responsible for miscarriages and aneuploid offspring in humans. However, recent findings indicate that the situation is not as straightforward and Rb fusions may also have an impact on the structure of the genome in germ cells, which could lead to functional and evolutionary consequences [203]. Indeed, Rb fusions can significantly change the 3D arrangement of chromatin in spermatocytes, potentially leading to new interactions between domains and exposing them to different regulatory environments that may impact gene expression and regulation, having implications for both fertility and evolution [167]. Additionally, the redistribution of COs across chromosomal arms in Rb mice was consistent with the “suppressed recombination” model, showing low recombination rates at Rb fusions, also affecting both chromosomal axis length and recombination [203].

This results in the existence of a gametic barrier due to the fixation of chromosomal rearrangements in a population, which allows for a genetic distinction between species. Genome sequencing analysis has indicated that these evolutionary rearrangements are much more numerous than initially estimated [204,205]. An example can be seen in the human and chimpanzee genomes, which were initially estimated to differ only in 9 chromosomal inversions and one fusion in 1980 [206,207], but later, in 2005, 93 supplementary evolutionary rearrangements were described, ranging from 12 kb to 1 Mb [208]. It has been now shown that human chromosome 2 was formed by the head-to-head fusion of two ancestral chromosomes that remained separate in other primates, corresponding to current chimpanzee chromosomes 12 and 13 [209].

In this sense, more than 245 large rearrangements including translocations, inversions, fusions, and deletions have been identified in the divergence between mice and humans [210], and several duplications involved in human-specific adaptive qualities have been recently characterized between the human and non-human primate genome [211].

##### Microchromosomes

Microchromosomes—chromosomes that are smaller in size than a typical chromosome—refer to chromosomes that have undergone structural changes, such as deletions, duplications, or inversions, resulting in a smaller size than normal, and sometimes a proper NE will form [87]. These changes can lead to a loss of genetic material. Micro chromosomes are not found in humans but are highly expressed in normal cells from birds and reptiles.

Bird and reptile karyotypes are striking in their differences, as in addition to normal-size mammalian chromosomes, they are generally characterized by the presence of well-conserved tiny microchromosomes, but little is known about the progression of meiosis in them. These are gene-rich and highly conserved between species of birds and reptiles. It seems that levels of DNA double-strand break (DSB) formation in reptiles are lower compared to mammals, suggesting that low recombination rates are a distinctive feature of reptiles [212,213]. Oppositely, micronuclei tend to clump into a central compartment at interphase and during mitosis and meiosis, leading to stronger inter-chromosomal interactions between microchromosomes rather than macrochromosomes, probably facilitating homology search and DSB formation that is maintained even in germ cells [212]. This suggests functional coherence. Many translocations of micronuclei and fusions with each other or with larger chromosomes have taken place independently in different clades, such as turtles, snakes, and lizards, or others have been lost, leading to diversity and speciation. In mammals, they have completely disappeared. The work by Waters and colleagues [212] has shed light on the contribution of micronuclei in evolution by comparing the genomes of different birds and reptiles, as well as mammals and amphioxus, providing evidence that microchromosomes are highly conserved ancient animal chromosomes, whereas macrochromosomes have undergone multiple lineage-specific rearrangements, especially in mammals, probably due to its higher level of DSB formation. 

##### Whole Genome Duplications and Gain in Chromosomes

Beyond rearrangements of genomic segments throughout evolution, large-scale genomic duplication may occur, but more rarely, involving a macroscopic portion of the genome or even the complete genome, also called Whole Genome Duplications (WGDs). 

WGD has a significant impact on an organism’s physical characteristics, and it is highly likely that it causes infertility and reduces the organism’s fitness, which can negatively impact the organism’s short-term survival [213]. Therefore, most WGD events do not result in viable outcomes, and they do not become fixed in the population. However, under specific conditions, WGD can provide an immediate evolutionary advantage in extreme environmental conditions [214,215]. It is now understood that WGD has played a significant role in evolution. WGD can reduce the risk of extinction for the affected lineage by alleviating its intrinsic disadvantages, and it can also help boost the biological fitness of the organism or create new species. This is because ancient WGDs often occurred during periods of environmental turmoil or extinction events [215,216]. Such advantages could explain why so many cancers present one to several rounds of WGD [15,213,215].

While WGD events are relatively common in plants, such as Arabidopsis, and have also been observed in the yeast Saccharomyces [217,218], identifying WGD pairs or quartets in vertebrates is more difficult. It is now widely accepted that two rounds of WGD happened at the origin of the vertebrate lineage about 500–550 million years ago [219,220] (Figure 2). However, how these global-scale WGD events affected the gene regulatory network is not yet fully understood in cancer cells. It has been proposed that a main vertebrate, WGD played a crucial role in the organization of multicellularity by influencing the interaction of proteins, the selection and retention of genes, and the balance of gene dosage [221]. 

Many tumors present rounds of WGD but often stabilize to a near 3N karyotype [15]. Some genes are selected to offer advantages. Interestingly, genes that have undergone duplication are three times more likely to be involved in cancer and autosomal dominant diseases, as they are more susceptible to dominant deleterious mutations [222]. Duplicated genes have stricter requirements for maintaining proper dosage balance [223], are normally involved in signaling, development, and transcriptional regulation, and are represented in gene ontology categories associated with an organism’s level of complexity [222,224,225]. Therefore, duplicated genes resulting from stabilized WGD tend to be essential, evolutionarily conserved genes and are related to increased redundancy and complexity, allowing for advantage under stressful or disturbed environments compared to non-WGD organisms [219,224]. This parallelism between WGD in evolution and the high incidence of WGD in cancer both strive for increasing fitness to overcome non-WGD. But it is also possible that the high incidence of cancer in the human population due to WGD is caused by adverse and polluted environmental conditions. However, further analysis is needed to fully understand the molecular mechanisms responsible for the advantage of WGD genomes over non-duplicated ones in cancer or evolution.

##### Chromothripsis

Traditionally, studies on evolution have treated genome alterations as the result of many incremental single steps. However, recent research has discovered that genomes can be altered in a single, large catastrophic event, leading to complex genomic rearrangements. This phenomenon is called chromothripsis [17]. Chromothripsis occurs in a single cell and results in the breaking and rearranging of one or multiple segments of chromosomes in a random manner (Figure 1). It was initially discovered in tumors [226], but it has also been identified in individuals with congenital malformations, developmental disorders, or seemingly balanced chromosomal rearrangements [227]. However, it has also been described in healthy individuals [228]. Furthermore, chromothripsis may also have a role in evolution as a source of new genetic combinations. Different hypotheses have been proposed, such as the idea that derived chromosomal rearrangements can restrict gene flow through suppression of recombination or facilitate rapid changes in patterns of genes unrelated to recombination suppression. Chromothripsis could also lead to the formation of new gene linkage blocks and new chimeric genes, as well as disruption of the cis-regulatory machinery for gene expression [229]. One proposed explanation for the connection between the various causes of chromothripsis and the limited scope of genomic changes it causes is that the affected chromosome(s) may be placed in a micronucleus [87,90]. The formation of micronuclei can happen due to a failure in chromosome segregation but also can be caused by various types of stress during any stage of the cell cycle, and it can persist for multiple cell cycles before being eliminated or incorporated back into the main nucleus [90].

#### 7.3.5. Repetitive DNA Elements as Contributors for Chromosome Evolution and Speciation

To fully understand how mammalian genomes are shaped and how speciation occurs, it is important to consider the structural organization of genomes. Research has shown that certain breakpoint regions tend to cluster in specific genomic features, such as duplications or repetitive elements [230,231], tandem repeats [232,233], and transposable elements [232,234]. Changes in the presence or length of these DNA elements can alter the chromatin state, which in turn can affect gene expression [3,197]. In an interesting paper published by Capilla et al. [6] on rodents, a family characterized by high chromosomal variability between clades, the authors found an association between evolutionary breakpoint regions and active chromatin state landscapes.

A repetitive DNA element that plays a role in driving genome evolution is the large tandem repeat known as satellite DNA (satDNA) [235]. satDNA has also been linked to various genetic disorders, such as cancer and developmental abnormalities [236]. The presence of satDNA remains a prevalent concern for various species, including both vertebrates and invertebrates. To establish comparative studies of genome divergences, satDNA is still an ongoing issue in sequencing techniques due to genome assembly and annotation. Recent studies have shown that satDNA can vary in abundance, size, and organization within a single genome, and it can also differ considerably among closely related species [235]. This dynamic behavior has led to speculations about the role satDNA plays in genome plasticity and chromosome rearrangements. Some studies suggest that satDNA could act as a source of genetic variation and evolution through mechanisms such as non-identical CO, replication errors, and non-allelic homologous recombination. These mechanisms could result in changes in the number and reorganization of chromosomes and genetic diversity [235,237]. 

Transposable elements, also known as transposons, play a significant role in chromosome evolution and speciation. They can act as drivers of DNA breaks and changes in chromatin conformation, both of which can compromise genomic stability [238]. However, they can also promote adaptability in a population through changes in gene expression or rapid chromosome restructuring [239,240]. An example of this is the gibbon genome, where the insertion of the retro-transposon LAVA in genes involved in cell cycle progression and chromosome segregation has led to a high rate of chromothripsis-related rearrangements and the emergence of different gibbon lineages with highly rearranged chromosomes [241,242]. Furthermore, the ZF domain from PRDM9 tends to recognize transposable elements in mammals. However, further research is needed to completely understand the role of repetitive DNA elements in the evolution of genomes [243].

#### 7.3.6. Telomeres

Telomeres have also been described as a driver for evolution. An interesting study reveals a unique feature of marsupial meiosis, a non-traditional model mammal, the detection of alternative lengthening of telomeres (ALT) during prophase I spermatogenesis [244]. They propose a telomeric elongation model in dunnarts, a family of marsupials native to Australia and New Guinea, that implies telomerase activity, telomere transcription, and alternative lengthening of telomeres (ALT) [245]. Opposite, telomere length remains stable through spermatogenesis or oogenesis in humans, mice, or any other mammal [246,247,248,249]. The open chromatin state of dunnart telomeres is coupled with heterologous telomeric associations in early pachytene, which supports the homologous recombination-based mechanism in ALT mediated by RPA and RAD51, searching and inducing inter-telomere recombination [212,250,251]. Interestingly, ALT was initially described in cancer cells, but further than in marsupial meiosis, it has also been detected in non-cancer pluripotent stem cells to maintain long telomeres that are heterogeneous in size [252,253]. This suggests that the mechanisms used to maintain long, heterogeneous telomeres in evolution may also be present in cancer. Supporting this idea, dasyurids have a genetic predisposition to lymphomas [254]. Then, further exploration of ALT for telomere homeostasis in dasyurids in the germline could provide new insight into the high incidence of tumors reported in this clade and establish a correlation. 

## 8. Consequences of Chromosomal Instability Other Than Cancer in Humans

A variety of genetic diseases associated with CIN has been described. Next, we detail some of them.

### 8.1. Infertility

The association between fertility and chromosomal instability is complex and multi-factorial. CIN can lead to infertility in several ways. For example, abnormal chromosomes can cause problems during meiosis, leading to the production of aneuploidy sperm or eggs. If these sperm or eggs are used in fertilization, the resulting embryos will have chromosomal abnormalities, leading to miscarriage or developmental problems. CIN can also lead to infertility affecting the mitotic self-renewing cells from the germ line, which ultimately give rise to the gametes. Inversion, as an important structural aberration, has been proven to be associated with male infertility [255]. However, although carriers with inversion of chromosome 10 have a high risk of infertility or recurrent miscarriages, they might still produce healthy offspring [256]. Another example is the inversion of chromosome 9 in humans, which is present in 2.6% of the population and not significantly associated with infertility or recurrent pregnancy loss [257]. One possible explanation for this is the shortened homologous region is not affected by the inversion, which reduces recombination levels between homologous chromosomes, thereby ensuring correct chromosome segregation. With the use of new genome sequencing techniques, we are now able to explore the complexity of chromosomal abnormalities in recurrent miscarriage, providing a deeper understanding of the underlying causes and potential targeted treatment options.

### 8.2. Rare Diseases

Rare diseases are defined as diseases that affect a small percentage of the population. Many rare diseases are caused by genetic mutations, and some may be related to CIN. Some specific syndromes are associated with CIN.

#### 8.2.1. Impairment of DNA Damage Repair Pathways 

Fanconi anemia (FA) is a recessive disease that is associated with growth retardation, congenital malformations, bone marrow failure, and a high risk of cancer. FA is a model syndrome of genome instability and is caused by a deficiency in DNA interstrand crosslink repair resulting in chromosome breakage. Cancer derived from FA presents a high number of structural variants [258]. FA is caused by biallelic mutations in at least 12 different genes, some of which form a nuclear core complex that involves the ubiquitination of the central FANCD2 protein. This process promotes the accumulation of FANCD2 in nuclear repair foci, along with BRCA1, BRCA2, and RAD51. Biallelic mutations in BRCA2 and FANCD2 can lead to a severe FA-like phenotype. The *FA* genes and *FANC2* are involved in DNA repair functions that are associated with the resolution of DNA crosslinks and stalled replication forks [258]. Although genetic instability caused by mutations in the *FANC* genes can be detrimental for most FA patients, around 20% appear to benefit from it, as it increases the chance of somatic reversion of their constitutional mutations. This can lead to improved bone marrow function through intragenic crossover, gene conversion, back mutation, and compensating mutations [258,259].

Another rare disease associated with DNA repair is Bloom syndrome, a genetic disorder that leads to developmental abnormalities and an increased risk of developing cancer. It is caused by mutations in the *BLM* gene, which is involved in the repair of DNA damage. 

Another rare disease is the Nijmegen breakage syndrome, a genetic disease that leads to increased susceptibility to infections, developmental delays, and an increased risk of cancer. It is caused by mutations in the *NBS1* gene, which is also involved in the repair of DNA damage. 

#### 8.2.2. Germline SV and CCR

Structural variants (SVs) represent 0.1% of variants [48] but account for 4 to 11.2% of deleterious coding alleles, a disproportionate contribution highlighting the major role of chromosomal instability in human disease [48]. Furthermore, SVs are responsible for 25–29% of all rare protein-truncating events per genome [49], making germline CIN an underestimated cause of rare diseases. Importantly, autism and schizophrenia were recently linked with specific SVs [48,260,261,262].

In children, much more than in adults, fusion proteins play an important role in driving tumorigenesis, and many different fusions have been identified as potential driver events in pediatric CNS neoplasms [51]. In pediatric cancer, 11% of known clinically relevant fusions detected at a high confidence level are germline [263].

Because of the tremendous advances obtained in sequencing tools, complex genomic rearrangements are also discovered to be an underestimated cause of rare diseases [264].

## 9. Concluding Remarks 

Investigating and understanding the high degree of plasticity of genomes has altered our perception of how genomes originate, remain stable, and evolve in cancer cells and during the speciation process. The development of new multidisciplinary approaches that combine sequencing technologies (e.g., long reads, single-cell DNA sequencing, etc.), bioinformatic tools, genomic approaches, and experimental methods have made it possible to study the functional and structural basis of the genomes of various organisms, strengthening the links between genome expression, genome stability, and 3D genome architecture. Nowadays, we are closer to being able to predict the consequences in developmental biology, such as cancer, fertility, and evolution, due to altered genomes, as reproducible patterns are revealed. It is common to find new studies focusing on the dynamics and plasticity of genome architecture within the framework of integrative analysis, its causes and consequences, and finding similarities between processes such as cancer evolution and macroevolution leading to speciation, with the main difference being the time scale. It is clear that the importance of genome-level events emphasizes the need for a new conceptual framework that integrates information based on genome organization. Alternatively, the spatial organization and folding of chromosomes within the nucleus play a crucial role in regulating gene expression, and this has important ramifications for evolution or the development of cancer.

Lessons from tumor evolution and macroevolution can be learned and translated from one to the other. In particular, cancer evolution represents a promising avenue for investigating macroevolutionary processes. The question of the gradualism model is now interrogated as the only mode of evolution, and new data suggests that a punctual burst of genomic instability leading to abrupt changes is indispensable to creating evolution and adaptation. The role of WGD and errors leading to micronuclei formation are, in both cases, important drivers of evolution and the generation of genomic chaos.

Here, we have summarized how different large-scale chromosome reorganizations take place and their consequences. From understanding the factors involved to conducting mechanistic and functional studies, we will gain new insights into the mechanisms responsible for these processes, providing an overview of similarities and differences in their sequential evolution steps.

## Data Availability

Not applicable.
